# Comorbid Diabetes Mellitus Contributes to Residual Overactive Bladder After Transurethral Surgery for Benign Prostatic Hyperplasia

**DOI:** 10.1111/iju.70080

**Published:** 2025-04-24

**Authors:** Koji Miki, Keisuke Kiba, Daisuke Maenosono, Yasunori Akashi, Mamoru Hashimoto, Yutaka Yamamoto, Kazutoshi Fujita, Akihide Hirayama

**Affiliations:** ^1^ Department of Urology Kindai Nara University Faculty of Medicine Ikoma Japan; ^2^ Department of Urology Kindai University Faculty of Medicine Osakasayama Japan

**Keywords:** benign prostatic hyperplasia, diabetes mellitus, lower urinary tract symptom, metabolic syndrome, overactive bladder

## Abstract

**Objectives:**

This study aimed to investigate the factors associated with residual overactive bladder after transurethral surgery for benign prostatic hyperplasia.

**Methods:**

This study retrospectively analyzed male patients aged 50 years or older who underwent transurethral surgery for benign prostatic hyperplasia with overactive bladder between January 2014 and December 2022. The subjects were divided into poor and good responder groups based on the presence of overactive bladder after surgery. Preoperative background factors, the International Prostate Symptom Score, Overactive Bladder Symptom Score, free uroflowmetry, pressure flow study, and subjective and objective findings at 3, 6, and 12 months after surgery were compared between the two groups. Furthermore, multivariate analysis was performed to identify risk factors associated with residual overactive bladder after surgery.

**Results:**

Sixty‐seven patients met the inclusion criteria. Among them, 18 and 49 patients were categorized as poor and good responders, respectively. Compared with the good responders, the poor responders had a significantly higher prevalence of metabolic syndrome, particularly waist circumference ≥ 85 cm and history of diabetes mellitus treatment. In addition, the poor responders had a higher preoperative maximum flow rate and lower bladder outlet obstruction index in the pressure flow study compared with the good responders. Multivariate analysis identified the history of diabetes treatment and the lower bladder outlet obstruction index as predictive factors for residual overactive bladder after surgery for benign prostatic hyperplasia.

**Conclusion:**

Our findings may improve the prediction of residual overactive bladder after surgery for benign prostatic hyperplasia.

## 1 Introduction

One of the main causes of lower urinary tract symptoms (LUTS) in elderly men is benign prostatic hyperplasia (BPH). LUTS due to BPH primarily arise from bladder outlet obstruction (BOO) and secondarily from bladder dysfunctions, such as increased sensory activity, decreased compliance, detrusor overactivity (DO), and detrusor underactivity [1].

Behavioral therapy and medication are typically used as initial treatment for BPH. However, surgical treatment is also indicated in the following cases: (1) the effect of medication is not enough, (2) there are moderate to severe symptoms, (3) complications such as urinary retention, urinary tract infections, hematuria, or bladder stones are present or suspected, or (4) there are hydronephrosis and increased creatinine levels. Surgical treatment for BPH commonly involves transurethral resection of the prostate, which remains the gold standard. Recently, enucleation techniques, laser surgery, water vapor energy therapy, lift techniques, and the like have become popular due to the development of various devices. Surgery for BPH with OAB often results in improvement in many cases, but we often experience cases whose OAB persists after surgery (residual OAB). About 30% of men with OAB continue to experience storage symptoms after surgery for relieving BOO [2].

In men with LUTS, metabolic syndrome (MetS), which is characterized by obesity, hypertension (HT), high blood glucose, and dyslipidemia, is considered a risk factor [3]. Some reports suggest that MetS promotes chronic ischemia and urothelial dysfunction by inducing oxystress, inflammation, and insulin resistance, which may lead to persist OAB [4]. There is possibility that MetS may influence the residual OAB after surgery, but this has not been extensively studied thus far. The aim of this study was to determine whether MetS contributes to residual OAB after transurethral surgery for BPH.

## 2 Methods

### 2.1 Patients

This study retrospectively analyzed 99 cases of elderly male patients aged 50 years or older who underwent transurethral surgery for BPH with OAB at Kindai University Nara Hospital between January 2014 and December 2022 and had at least 12 months of follow‐up after transurethral surgery. The exclusion criteria were cases with neurological disorders, spinal cord diseases, cerebrovascular accidents, or those who had undergone surgery or radiotherapy for bladder cancer or prostate cancer, as well as cases with insufficient data.

Age, body mass index (BMI), individual diagnostic items of MetS, prostate volume (PV), free uroflowmetry (UFM), pressure flow study (PFS), International Prostate Symptom Score (IPSS), Overactive Bladder Symptom Score (OABSS), and quality of life (IPSS‐QoL) score were assessed in all patients as a standard procedure.

The diagnostic criteria for OAB were an OABSS question number 3 (urinary urgency) score of at least 2 and a total score of at least 3 in accordance with the Japanese OAB guidelines [5].

The diagnosis of MetS was based on the Japan Society for the Study of Obesity diagnostic criteria [6]. The criteria were as follows: a waist circumference (WC) ≥ 85 cm or more and meeting two or more of the following three criteria: (1) serum triglyceride (TG) levels ≥ 150 mg/dL and/or high‐density lipoprotein cholesterol (HDL‐C) levels < 40 mg/dL, (2) systolic blood pressure ≥ 130 mmHg and/or diastolic blood pressure ≥ 85 mmHg, and (3) fasting plasma glucose (FPG) levels ≥ 110 mg/dL. Additionally, treatment with medication for high TG levels, low HDL‐C, HT, or DM was considered positive for each respective component.

In accordance with the EAU guidelines, the choice of procedure was left to the discretion of each urologist and patient. After surgery, a urethral catheter was inserted until the gross hematuria had almost disappeared [7]. Patients were discharged after catheter removal if they could urinate without experiencing urinary retention. Patients were evaluated at 3, 6, and 12 months using after surgery IPSS, IPSS‐QoL, OABSS, and UFM. Surgical complications were assessed using the Clavien‐Dindo classification [8]. In cases of residual OAB, additional drug treatment for OAB could be given if the patient so wishes.

The transurethral surgeries performed included transurethral resection of the prostate in saline (TURis), transurethral enucleation with bipolar energy (TUEB), and photoselective vaporization of the prostate (PVP).

TURis was performed using a bipolar electrosurgical system (UES‐40S, Olympus, Tokyo, Japan), a 26‐Fr continuous flow resectoscope with a 12°telescope (Olympus); the prostate adenoma was resected using the standard high‐frequency electrode loop, and the prostate chips were evacuated [9].

TUEB was performed using a bipolar electrosurgical system (UES‐40S, Olympus), a 26‐Fr continuous flow resectoscope with a 12°telescope (Olympus), a specially designed, high‐frequency electrode loop with a spatula, and a high‐frequency electrode loop (Olympus). The procedure was performed according to previously reported techniques [10]. After enucleation, the prostate adenoma was resected using the standard high‐frequency electrode loop, and the prostate chips were evacuated. Transurethral morcellation was not performed.

PVP was performed using a GreenLight 120 W HPS system (American Medical System Inc., Minnetonka, MN, USA). A laser fiber was inserted through the working channel of a continuous double‐flow 23‐Fr resectoscope with normal saline irrigation. The procedure was performed according to previously reported techniques, such as the Malek technique, anterior start technique, and Basel technique [11].

### 2.2 Statistical Analysis

The group that met the Japanese OAB diagnostic criteria preoperatively and at 12 months postoperatively was classified as PR, and the group that did not meet the OAB diagnostic criteria at 12 months postoperatively was classified as GR. The following parameters were compared between the two groups: age; WC; BMI; MetS parameters (history of HT, fasting hyperglycemia or DM treatment, dyslipidemia); medication history; PV; postvoid residual urine; IPSS; IPSS voiding subscore; IPSS storage subscore; OABSS; IPSS‐QoL; UFM parameters; PFS parameters; and postoperative IPSS, OABSS, and UFM parameters at each follow‐up visit, as well as postoperative complications. Furthermore, multivariate analysis was performed to identify risk factors associated with residual OAB.

Results were compared between the two groups using the Mann–Whitney *U*‐test for continuous variables, and the chi‐square test and Fisher's exact test for qualitative variables. Comparisons between baseline and each follow‐up visit were performed using the paired *t*‐test. Additionally, a multivariate logistic regression analysis was conducted to examine the predictive factors for residual OAB after surgery for BPH. Univariate analysis was performed for variables such as age, PV, WC, history of HT, DM treatment, dyslipidemia, DO, and BOOI. Multivariate analysis was then performed using variables that showed significant differences in the univariate analysis. The cutoff values for postoperative OAB persistence and improvement in OAB were determined using receiver operating characteristic (ROC) curve analysis for BOOI. All tests were two‐sided, and a *p*‐value of < 0.05 was considered significant. All statistical analyses were performed using SPSS version 23 (IBM, Armonk, New York).

## 3 Results

Of the 134 men aged 50 years or older who underwent transurethral surgery for BPH, 99 (73.9%) had OAB before surgery. Of these, 1 patient with cerebrovascular disease, 2 patients with previous treatment for prostate or bladder cancer, and 29 patients with missing data were excluded from this analysis. The analysis therefore included the remaining 67 (67.7%) patients. Then, 11 of the 18 patients in the PR group were treated with beta‐3 agonist and/or antimuscarinic drugs.

Table 1 presents the characteristics and subjective and objective findings of the two patient groups. The PR and GR groups consisted of 18 (27%) and 49 (73%) patients, respectively. Regarding background factors, the PR group had a higher prevalence of MetS, particularly WC ≥ 85 cm and impaired glucose tolerance (FPG ≥ 110 mg/dL and/or a history of DM treatment), compared to the GR. Furthermore, while the proportion of FPG ≥ 110 mg/dL showed no significant difference between the two groups, the prevalence of a history of DM treatment was significantly higher in the PR group. There were no significant differences in preoperative medications or surgical procedures between the groups. Regarding postoperative complications, there was one case (6%) of Clavian‐Dindo classification I (chronic urinary tract infection) and one case (6%) of II (residual adenoma) in PR, and two cases (4%) of Clavian‐Dindo classification I (chronic urinary tract infection in two cases) and four cases (8%) of II (urethral stricture in two cases, residual adenoma in one case and bladder stone disease in one case) in GR, but there were no significant differences between the two groups and no complications above III.

**TABLE 1:** Preoperative characteristics and preoperative data.

Variables	Poor responder	Good responder	*p*
	(*n* = 18, mean ± SD)	(*n* = 49, mean ± SD)	
Age (years)	74.2 ± 4.5	72.1 ± 5.7	0.169
BMI	25.8 ± 11.8	23.0 ± 3.2	0.994
PV (mL)	64.2 ± 39.5	65.9 ± 28.9	0.38
MetS	11 (61%)	15 (31%)	0.046
Waist ≧ 85 cm	15 (83%)	27 (55%)	0.043
Hypertension	13 (72%)	31 (63%)	0.495
110 mg/dL ≧ or DM	14 (77%)	11 (22%)	0.001
FPG 110 mg/dL≧	3 (17%)	4 (8%)	0.323
DM	9 (50%)	7 (14%)	0.004
TG ≧ 150	4 (22%)	10 (20%)	0.871
HDL‐C < 40	7 (39%)	13 (27%)	0.33
IPSS			
Total score	20.3 ± 6.5	24.4 ± 6.5	0.019
Storage score	10.2 ± 2.9	10.3 ± 3.0	0.994
Voiding score	10.1 ± 5.2	14.1 ± 4.0	0.004
IPSS‐QoL	4.8 ± 0.9	5.1 ± 0.9	0.171
OABSS			
Total score	9.0 ± 2.8	8.4 ± 2.2	0.411
Daytime frequency	1.0 ± 0.3	1.1 ± 0.6	0.55
Nocturia	2.3 ± 0.9	2.5 ± 0.6	0.606
Urgency	3.3 ± 1.4	3.5 ± 1.0	0.93
Urge incontinence	2.6 ± 1.5	1.4 ± 1.4	0.004
Preoperative medications history			
Alpha‐brocker	15 (83%)	42 (91%)	1
5 alpha‐reductase inhibitors	8 (44%)	27 (55%)	0.582
Phosphodiesterase type 5 inhibitor	3 (17%)	9 (18%)	1
Medications for OAB	4 (22%)	9 (18%)	0.736
Beta‐3 agonist	4 (22%)	9 (18%)	0.736
Antimuscarinic drugs	2 (11%)	1 (2%)	0.174
Surgical method			
TURis	3 (17%)	5 (10%)	0.672
TUEB	8 (44%)	28 (57%)	0.415
PVP	7 (39%)	16 (33%)	0.773
Clavian‐Dindo			
I≧	2 (12%)	6 (12%)	0.899
I	1 (6%)	2 (4%)	0.797
II	1 (6%)	4 (8%)	0.72
III≧	0 (0%)	0 (0%)	

Abbreviations: BMI, body mass index; DM, diabetes mellitus; FPG, fasting plasma glucose; HDL‐C, high‐density lipoprotein‐cholesterol; IPSS, international prostate symptom score; IPSS‐QoL, IPSS‐quality of life; MetS, metabolic syndrome; OABSS, overactive bladder symptom score; PV, prostate volume; PVP, photoselective vaporization of the prostate; TG, triglycerides; TUEB, transurethral enucleation with bipolar; TURis, transurethral resection of prostate in saline.

Table 2 presents the preoperative uroflowmetry results. The PR group had a higher Qmax at PFS and a lower BOOI compared with the GR group.

**TABLE 2:** Preoperative urodynamic study.

Variables	Poor responder	Good responder	*p*
	(*n* = 18, mean ± SD)	(*n* = 49, mean ± SD)	
UFM			
Qmax (mL/s)	5.7 ± 2.8	3.9 ± 2.2	0.281
VV (mL)	119.7 ± 78.4	134.5 ± 62.8	0.473
PVR (mL)	120.2 ± 118.4	141.6 ± 134.0	0.483
Urodynamic study			
PFS			
First desire to void (mL)	147.4 ± 58.9	141.9 ± 66.7	0.476
Normal desire to void (mL)	221.8 ± 79.0	200.0 ± 84.4	0.301
Maximum desire to void (mL)	288.4 ± 91.8	276.8 ± 106.3	0.545
Detrusor overactivity (mL)	8 (44%)	26 (53%)	0.735
PdetQmax (cm H_2_O)	75.8 ± 35.4	91.8 ± 31.4	0.167
Qmax (mL/s)	5.84 ± 3.2	4.0 ± 2.2	0.029
BOOI	63.4 ± 34.4	85.4 ± 30.6	0.036
BCI	104.7 ± 36.6	111.8 ± 27.1	0.804

Abbreviations: BCI, bladder contractility index; BOOI, bladder outlet obstruction index; PFS, pressure flow study; PVR, postvoid residual urine volume; Qmax, maximal urine flow rate; UFM, Free uroflowmetry; VV, voiding volume.

**FIGURE 1:**
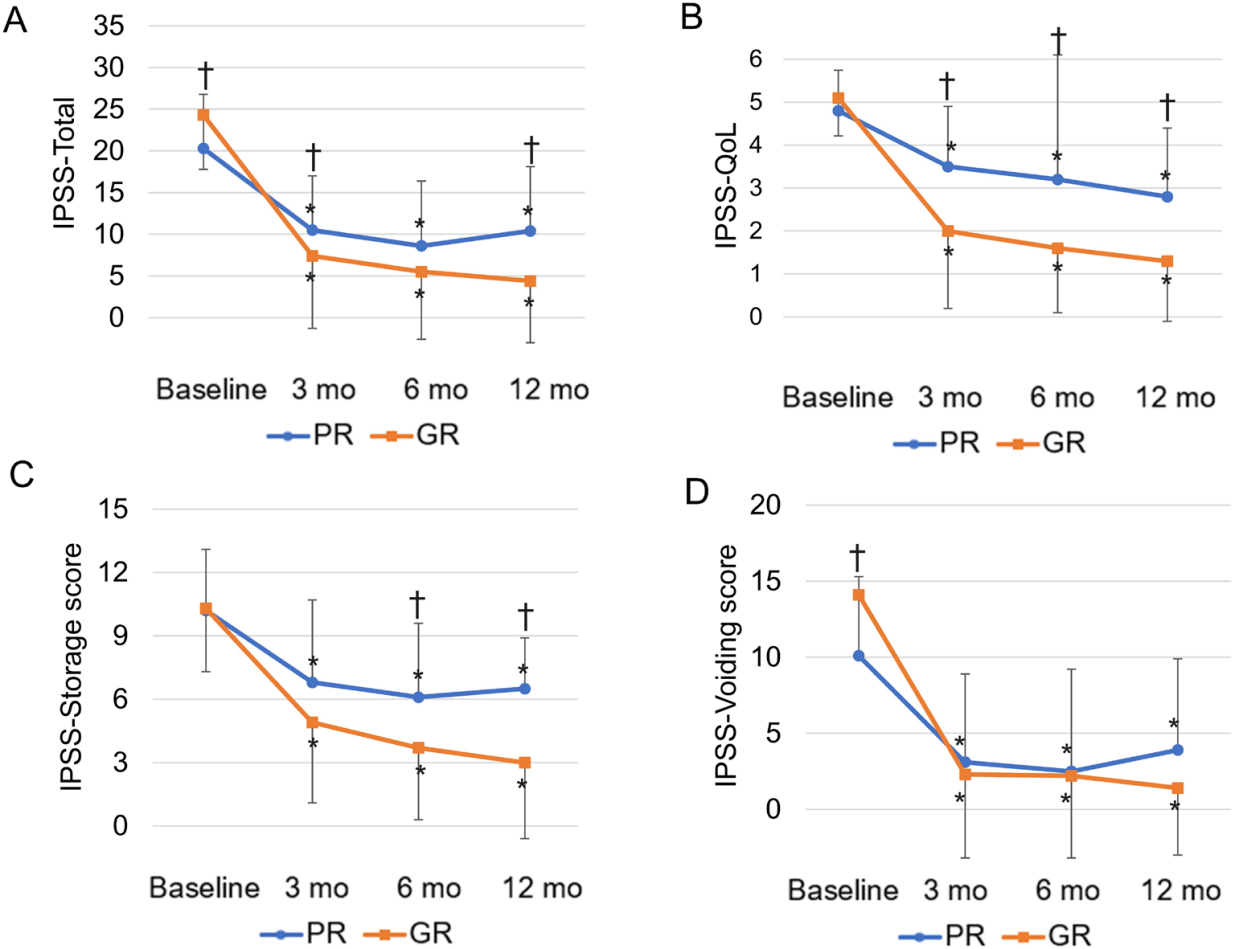
Mean baseline and postoperative values at different points of follow‐up are shown for (A) International Prostate Symptom Score (IPSS), (B) IPSS—Quality of Life (IPSS‐QoL), (C) IPSS storage symptom score, (D) IPSS voiding symptom score. *At each follow‐up visit, significant differences were found from the value at baseline. † A significant difference between two groups was found in the change in each parameter from baseline to each follow‐up visit.

Figure 1 shows the preoperative and postoperative history of IPSS. Both groups showed significant improvements in IPSS total score, IPSS‐QoL score, IPSS voiding subscore, and IPSS storage subscore at each postoperative time point compared with preoperative values. In the comparison between the two groups, the preoperative IPSS total score and IPSS voiding subscore were higher in the GR group. Postoperatively, the PR group exhibited poorer improvement in the IPSS total score and IPSS storage subscore. There was no significant difference between the two groups in terms of improvement in the IPSS voiding subscore.

**FIGURE 2:**
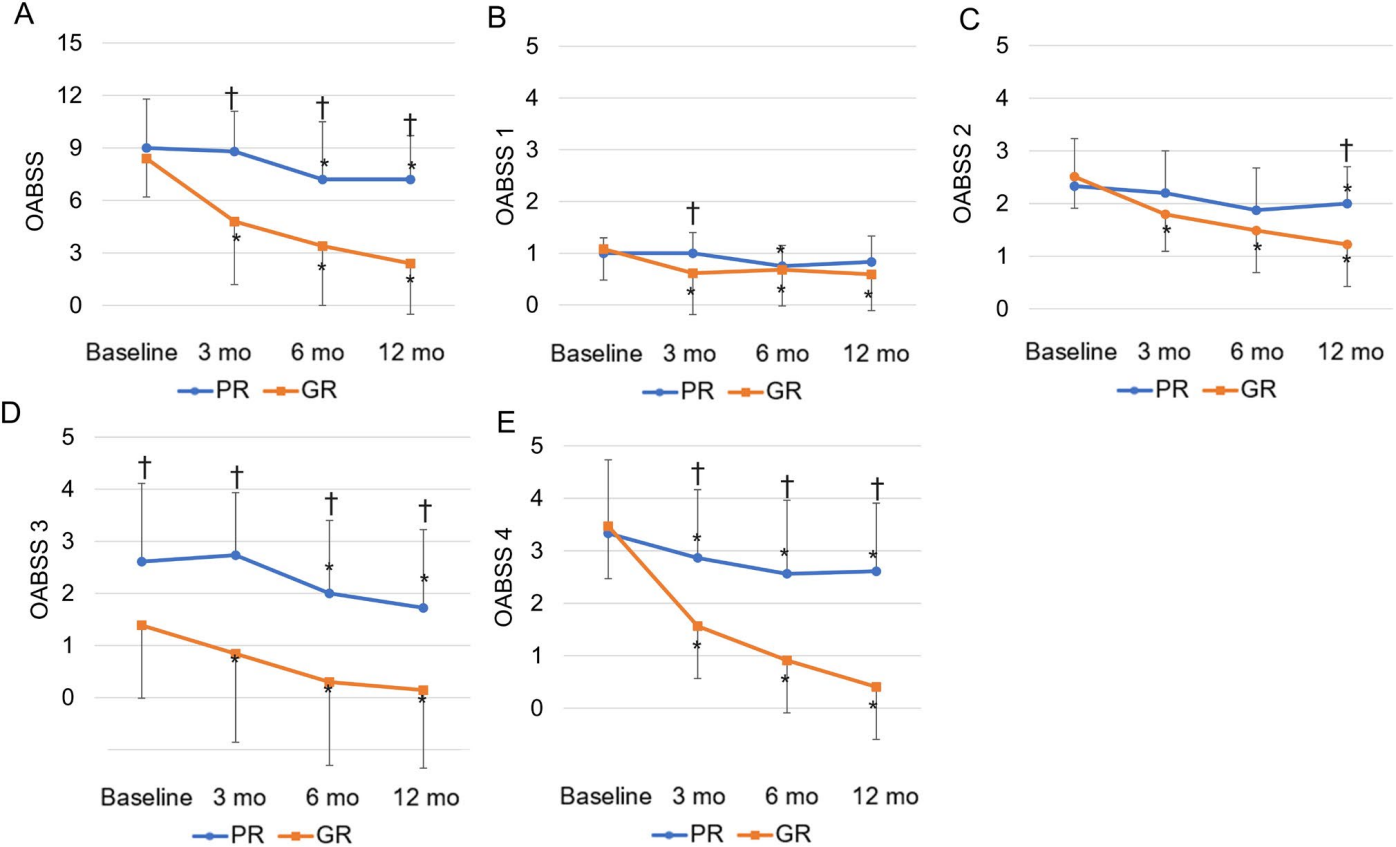
Mean baseline and postoperative values at different points of follow‐up are shown for (A) overactive bladder symptom score (OABSS), (B) OABSS 1 (daytime frequency), (C) OABSS 2 (nocturia), (D) OABSS 3 (urgency), and (E) OABSS 4 (urge incontinence). *At each follow‐up visit, significant differences were observed compared with the baseline value. † A significant difference between two groups was found in the change in each parameter from baseline to each follow‐up visit.

Figure 2 shows the preoperative and postoperative history of OABSS. There were no significant differences in the preoperative OABSS total score and each factor between the two groups. Postoperative OABSS total score, urgency, and urge incontinence showed significant improvement in both groups compared with preoperative values, but PR showed less improvement postoperatively compared with GR. Daytime frequency and nocturia improved significantly at each time point in GR, whereas PR did not show sustained improvement.

**FIGURE 3:**
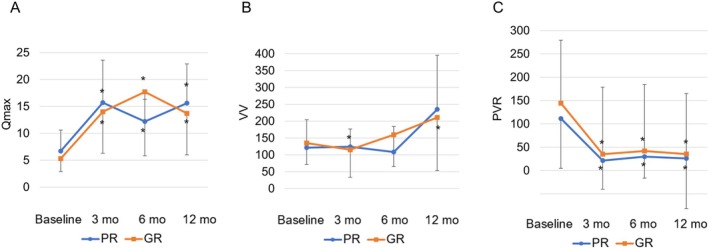
Mean baseline and postoperative values at different points of follow‐up are shown for (A) maximal urine flow rate (Qmax, mL/s), (B) voiding volume (VV, mL), and (C) postvoid residual urine volume (PVR, mL). *At each follow‐up visit, significant differences were observed compared with the baseline value. † A significant difference between two groups was found in the change in each parameter from baseline to each follow‐up visit.

Figure 3 shows the preoperative and postoperative history of UFM. Both groups showed significant improvements in each parameter at each postoperative time point compared with preoperative. The comparisons between the two groups did not reveal any significant differences at each time point.

Table 3 presents the predictors of residual OAB after surgery for BPH. Univariate analysis revealed significant differences in WC ≥ 85 cm, history of diabetes treatment, and BOOI. The multivariate analysis identified a history of diabetes treatment (OR 14.491, 95% CI 2.405–87.323, *p* = 0.004) and lower BOOI (OR 0.96, 95% CI 0.931–0.991, *p* = 0.011) as predictive factors for residual OAB after surgery.

**TABLE 3:** Univariate and multivariate analysis of variables associated with residual postoperative OAB.

	Univariate analysis		*p*	Multivariate analysis		*p*
	OR	95% CI		OR	95% CI	
Age (years)	1.081	(0.972–1.202)	0.151			
PV (mL)	0.998	(0.981–1.016)	0.838			
Waist ≧ 85 cm	4.074	(1.044–15.896)	0.043	2.775	(0.557–13.822)	0.213
Hypertension	1.51	(0.462–4.931)	0.495			
DM	6	(1.767–20.369)	0.004	14.491	(2.405–87.323)	0.004
TG ≧ 150	1.114	(0.301–4.132)	0.871			
HDL‐C < 40	1.762	(0.563–5.512)	0.33			
DO	0.846	(0.273–2.626)	0.773			
BOOI	0.974	(0.951–0.997)	0.029	0.96	(0.931–0.991)	0.011

Abbreviations: BOOI, bladder outlet obstruction index; DM, diabetes mellitus; DO, detrusor overactivity; HDL‐C, high‐density lipoprotein‐cholesterol; PV, prostate volume; TG, triglycerides.

**FIGURE 4:**
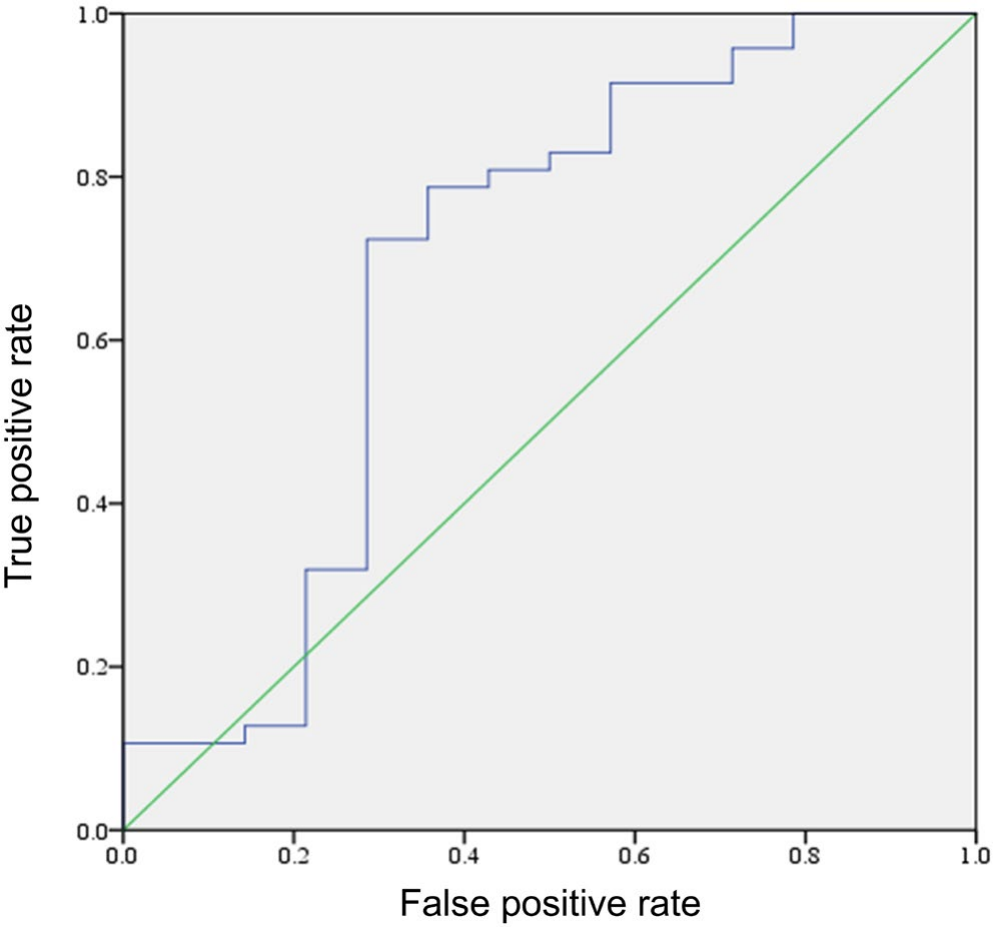
ROC curves of BOOI for predicting residual postoperative OAB. BOOI, bladder outlet obstruction index; ROC, receiver operating characteristic.

Additionally, analysis using the ROC curve showed that the BOOI value potentially associated with the risk of residual OAB after surgery had a cutoff value of 68.5 (sensitivity 72%, specificity 71%) (Figure 4).

## 4 Discussion

This study revealed that individuals treated for DM following transurethral surgery for BPH with OAB may be at risk of experiencing residual OAB symptoms. Currently, very few studies have documented a link between DM and persistent OAB symptoms following surgical intervention. The current findings may improve the prediction of remaining OAB symptoms following surgery for BPH.

Various factors are thought to contribute to the development of OAB in patients with DM, including inflammation, diabetic vasculopathy, and neuropathy [12, 13, 14]. Fater et al. also reported age, diabetic peripheral neuropathy (DPN), and duration of DM of more than 10 years as risk factors for increased severity of OAB in patients diagnosed with type 2 diabetes [15]. In this study, FPG ≥ 110 mg/dL was not a risk factor, and only a history of DM treatment was a risk factor for residual OAB after surgery. Patients with FPG ≥ 110 mg/dL may include prediabetic patients who do not meet the diagnostic criteria for DM, whereas patients with a history of DM treatment are patients who have been diagnosed and started on treatment for DM, and therefore may include many patients with the abovementioned factors involved in the development of OAB due to prolonged exposure to hyperglycemia. This suggests that patients with a history of DM treatment were at risk when compared to FPG ≥ 110 mg/dL. Such vascular and neuropathy of the bladder due to impaired glucose tolerance may not be ameliorated by the removal of BOO and may be the cause of residual OAB after surgery. Although patients with DPN were not included in this study, it is possible that OAB may remain postoperatively even in the absence of DPN. This may be related to the fact that DM not only induces systemic inflammation, but also microvascular and macrovascular complications, which may be involved in inducing impaired blood flow and chronic ischemia [3]. Further studies are needed to examine various factors, such as the presence or absence of diabetic complications, duration of disease, and treatment details.

Mauro et al. reported that WC ≥ 102 cm may serve as a risk factor for poor improvement in storage symptoms after BPH surgery [16]. Similarly, although multivariate analysis in our study did not reveal a significant difference, the proportion of patients with WC ≥ 85 cm was higher in the PR than in the GR, aligning with the above findings. Reports suggesting that MetS is a risk factor for OAB are not uncommon, with increased insulin resistance and obesity in MetS considered potential contributors to LUTS [17]. The mechanisms underlying these associations have been reported as follows: (1) Hyperinsulinemia is associated with increased sympathetic nervous system activity, which contributes to elevated smooth muscle tone in the prostate, potentially exacerbating LUTS independent of prostate enlargement. Additionally, hyperglycemia may increase cytosolic free calcium in smooth muscle cells and nerve tissue, possibly playing a role in sympathetic nervous system activation. (2) Obesity is characterized by visceral fat accumulation and dysregulation in the production of adipocytokines, which may increase the risk of metabolic abnormalities such as hyperglycemia, lipid metabolism disorders, and HT. These metabolic abnormalities may induce insulin resistance and associated inflammation, promote atherosclerosis, and trigger chronic inflammation or ischemia, which are believed to exacerbate LUTS [18, 19].

In BOO, the cycle of bladder stretching, high pressure, ischemia, and reperfusion is repeatedly triggered, with urethral resistance preventing urinary drainage, which gradually leads to various changes in the epithelium, nerves, and smooth muscle. Transurethral surgeries can improve this cycle by releasing BOO, which can improve voiding symptoms as well as storage symptoms [20]. In this study, GR also showed good improvement in both voiding symptoms and storage symptoms. However, it has also been reported that BOO can cause irreversible morphological changes, and OAB may remain after releasing BOO [21]. There have been scattered reports on the severity of BOO and improvement in postoperative LUTS. There are reports that a higher BOOI may be attributed to improved postoperative LUTS symptoms, with poor improvement in DO patients without BOO [22, 23]. In the present study, lower BOOI was associated with a risk of residual OAB after surgery, which is consistent with these reports. Furthermore, the cut‐off value of BOOI for predicting residual OAB, as calculated in the present study using ROC curves, was 68.5. As a rule, all patients in this study underwent UDS, and surgery was indicated for a BOOI of 40 or above. Thus, only patients with high BOOI were included in this study. Hence, the cut‐off values may change if patients with a BOOI below 40 are included. Preoperative DO was not a significant risk factor for residual OAB after surgery in this study. Several studies reported that preoperative DO does not affect the improvement of postoperative storage symptoms [24]. However, other studies reported that the presence of preoperative DO might be negatively associated with postoperative symptom improvement [25, 26, 27]. Further research is needed to examine whether the preoperative DO is a predictive factor for residual OAB.

In our study, PR had a lower severity of preoperative IPSS total score and voiding subscore and a greater degree of preoperative urgency compared to GR. They also had a lower Qmax at PFS. These may be features of residual OAB after surgery. Comparing the postoperative improvement in the two groups, the PR showed improvement in urgency and urge incontinence, and little improvement in daytime frequency and nocturia. However, not only voiding symptoms but also storage symptoms improved to some extent, suggesting that OAB caused by BOO was improved by surgery. Therefore, surgery might be aggressively considered in cases of BOO. OAB caused by DM or other causes may persist after releasing BOO and may be treated with additional postoperative medication, botulinum toxin injections, or sacral nerve stimulation.

Our study has some limitations. First, this was a retrospective study with a small sample size. In addition, the number of voidings or volume per micturition was not assessed using a voiding diary. Although the volume of single voiding was assessed by UFM at each time point, it is possible that some patients may not necessarily have sufficient urine storage. There was also a lack of personal information, such as alcohol or caffeine intake, which may influence urinary tract symptoms, and the possibility of de novo OAB, which may be a new occurrence after surgery but was not assessed in detail in this study.

In conclusion, a history of DM treatment and low BOOI may be associated with a higher incidence of OAB following surgery for BPH. The current findings may help predict residual OAB after surgery.

## Author Contributions

Koji Miki: Writing – original draft preparation. Keisuke Kiba: Conceptualization; writing – review and editing. Daisuke Maenosono: Data curation. Yasunori Akashi: Writing – review and editing. Mamoru Hashimoto: Writing – review and editing. Yutaka Yamamoto: Writing – review and editing. Akihide Hirayama: Supervision. Kazutoshi Fujita: Supervision.

## Ethics Statement

Ethical approval was provided by the Institutional Review Board of Kindai University Nara Hospital (No. 764) as instituted by the Declaration of Helsinki (Number 20–11).

## Consent

The patient consent to review the medical record was not required by the committee due to the retrospective nature of the study. All the data were anonymized and maintained with confidentiality.

## Conflicts of Interest

Kazutoshi Fujita is an Editorial Board member of International Journal of Urology and a co‐author of this article. To minimize bias, he was excluded from all editorial decision‐making related to the acceptance of this article for publication.

## References

[1] L. B. Lerner , K. T. McVary , M. J. Barry , et al., “Management of Lower Urinary Tract Symptoms Attributed to Benign Prostatic Hyperplasia: AUA GUIDELINE PART I‐Initial Work‐Up and Medical Management,” Journal of Urology 206, no. 4 (2021): 806–817. 34384237 10.1097/JU.0000000000002183

[2] A. A. Antunes , A. Iscaife , S. T. Reis , et al., “Can We Predict Which Patients Will Experience Resolution of Detrusor Overactivity After Transurethral Resection of the Prostate?,” Journal of Urology 193, no. 6 (2015): 2028–2032. 25583645 10.1016/j.juro.2014.12.095

[3] R. Chess‐Williams and D. J. Sellers , “Pathophysiological Mechanisms Involved in Overactive Bladder/Detrusor Overactivity,” Current Bladder Dysfunction Reports 18 (2023): 79–88.

[4] B. Peyronnet , E. Mironska , C. Chapple , et al., “A Comprehensive Review of Overactive Bladder Pathophysiology: On the Way to Tailored Treatment,” European Urology 75, no. 6 (2019): 988–1000. 30922690 10.1016/j.eururo.2019.02.038

[5] Y. Homma , M. Yoshida , N. Seki , et al., “Symptom Assessment Tool for Overactive Bladder Syndrome–Overactive Bladder Symptom Score,” Urology 68, no. 2 (2006): 318–323. 16904444 10.1016/j.urology.2006.02.042

[6] K. Yamagishi and H. Iso , “The Criteria for Metabolic Syndrome and the National Health Screening and Education System in Japan,” Epidemiol Health 39 (2017): e2017003. 28092931 10.4178/epih.e2017003 PMC5343105

[7] S. Gravas , J. N. Cornu , M. Gacci , et al., “EAU Guidelines on the Treatment and Follow‐Up of Non‐Neurogenic Male Lower Urinary Tract Symptoms Including Benign Prostatic Obstruction,” European Urology 5, no. 3 (2022): 30–49. 10.1016/j.eururo.2013.03.004 23541338

[8] D. Dindo , N. Demartines , and P. A. Clavien , “Classification of Surgical Com Plications: A New Proposal With Evaluation in a Cohort of 6336 Patients and Results of a Survey,” Annals of Surgery 240, no. 2 (2004): 205–213. 15273542 10.1097/01.sla.0000133083.54934.ae PMC1360123

[9] National Institute for Health and Care Excellence (NICE) , “[Internet] The PLASMA System for Transurethral Resection and Haemostasis of the Prostate,” (NICE Medical Technology Guidance MTG53, 2021), https://www.nice.org.uk/guidance/mtg53.

[10] C. Bebi , M. Turetti , E. Lievore , et al., “Bipolar Transurethral Enucleation of the Prostate: Is It a Size‐Independent Endoscopic Treatment Option for Symptomatic Benign Prostatic Hyperplasia?,” PLoS One 16, no. 6 (2021): e0253083. 34106986 10.1371/journal.pone.0253083 PMC8189479

[11] T. Kobayashi , N. Seki , Y. H. Song , and T. Dejima , “GreenLight HPS Laser 120 W vs. Diode Laser 300 W Vaporization of the Prostate for the Treatment of Benign Prostatic Hyperplasia in Japanese Patients: A Prospective, Single‐Center, Randomized Clinical Trial,” Lower Urinary Tract Symptoms 13, no. 1 (2021): 31–37. 32515894 10.1111/luts.12324

[12] M. A. Banakhar , T. F. Al‐Shaiji , and M. M. Hassouna , “Pathophysiology of Overactive Bladder,” International Urogynecology Journal 23, no. 8 (2012): 975–982. 22310925 10.1007/s00192-012-1682-6

[13] A. V. Sarma , J. K. Parsons , K. McVary , and J. T. Wei , “Diabetes and Benign Prostatic Hyperplasia/Lower Urinary Tract Symptoms—What Do we Know?,” Journal of Urology 182, no. 6S (2009): S32–S37. 19846144 10.1016/j.juro.2009.07.088

[14] O. Yamaguchi , M. Nomiya , and K. E. Andersson , “Functional Consequences of Chronic Bladder Ischemia,” Neurourology and Urodynamics 33 (2014): 54–58. 24292974 10.1002/nau.22517

[15] F. A. Khadour , Y. A. Khadour , W. Alhatem , and B. D. Al , “Risk Factors Associated With the Severity of Overactive Bladder Among Syrian Patients With Type 2 Diabetes,” Scientific Reports 14, no. 1 (2024): 16547. 39020001 10.1038/s41598-024-67326-w PMC11255225

[16] M. Gacci , A. Sebastianelli , M. Salvi , et al., “Central Obesity Is Predictive of Persistent Storage Lower Urinary Tract Symptoms (LUTS) After Surgery for Benign Prostatic Enlargement: Results of a Multicentre Prospective Study,” BJU International 116, no. 2 (2015): 271–277. 25597623 10.1111/bju.13038

[17] C. De Nunzio , W. Aronson , S. J. Freedland , E. Giovannucci , and J. K. Parsons , “The Correlation Between Metabolic Syndrome and Prostatic Diseases,” European Urology 61, no. 3 (2012): 560–570. 22119157 10.1016/j.eururo.2011.11.013

[18] H. Y. Ngai , K. S. Yuen , C. M. Ng , C. H. Cheng , and S. P. Chu , “Metabolic Syndrome and Benign Prostatic Hyperplasia: An Update,” Asian Journal of Urology 4, no. 3 (2017): 164–173. 29264226 10.1016/j.ajur.2017.05.001 PMC5717972

[19] E. Kassi , P. Pervanidou , G. Kaltsas , et al., “Metabolic Syndrome: Definitions and Controversies,” BMC 9 (2011): 48. 10.1186/1741-7015-9-48 PMC3115896 21542944

[20] M. M. Mostafa , A. Khallaf , M. Khalil , M. A. Elgammal , and A. Mahdy , “Efficacy and Safety of TURP, HoLEP, and PVP in the Management of OAB Symptoms Complicating BPH in Patients With Moderately Enlarged Prostates: A Comparative Study,” Canadian Urological Association Journal 17, no. 1 (2023): E1–E7. 36121889 10.5489/cuaj.7905 PMC9872828

[21] N. Singla and A. K. Singla , “Evaluation and Management of Lower Urinary Tract Symptoms After Outlet Surgery for Benign Prostatic Hyperplasia,” Current Bladder Dysfunction Reports 11, no. 3 (2016): 242–247. 32362986 10.1007/s11884-016-0376-1 PMC7194211

[22] Y. Tanaka , N. Masumori , N. Itoh , S. Furuya , H. Ogura , and T. Tsukamoto , “Is the Short‐Term Outcome of Transurethral Resection of the Prostate Affected by Preoperative Degree of Bladder Outlet Obstruction, Status of Detrusor Contractility or Detrusor Overactivity?,” International Journal of Urology 13 (2006): 1398–1404. 17083391 10.1111/j.1442-2042.2006.01589.x

[23] N. Masumori , R. Furuya , Y. Tanaka , S. Furuya , H. Ogura , and T. Tsukamoto , “The 12‐Year Symptomatic Outcome of Transurethral Resection of the Prostate for Patients With Lower Urinary Tract Symptoms Suggestive of Benign Prostatic Obstruction Compared to the Urodynamic Findings Before Surgery,” BJU International 105 (2010): 1429–1433. 19863522 10.1111/j.1464-410X.2009.08978.x

[24] V. W. Nitti , Y. Kim , and A. J. Combs , “Voiding Dysfunction Following Transurethral Resection of the Prostate: Symptoms and Urodynamic Findings,” Journal of Urology 157 (1997): 600–603. 8996367

[25] Y. R. Zhao , W. Z. Liu , M. Guralnick , et al., “Predictors of Short‐Term Overactive Bladder Symptom Improvement After Transurethral Resection of Prostate in Men With Benign Prostatic Obstruction,” International Journal of Urology 21, no. 10 (2014): 1035–1040. 24825248 10.1111/iju.12482

[26] A. A. Alberto , I. Alexandre , and T. R. Sabrina , “Can We Predict Which Patients Will Experience Resolution of Detrusor Overactivity After Transurethral Resection of the Prostate?,” Journal of Urology 193, no. 6 (2015): 2028–2032. 25583645 10.1016/j.juro.2014.12.095

[27] M. Creta , C. Collà Ruvolo , N. Longo , et al., “Detrusor Overactivity and Underactivity: Implication for Lower Urinary Tract Symptoms Related to Benign Prostate Hyperplasia Diagnosis and Treatment,” Minerva Urology and Nephrology 73, no. 1 (2021): 59–71. 32026666 10.23736/S2724-6051.20.03678-4

